# Revisiting thrombophilia testing: leveraging next-generation sequencing for precision in VTE management

**DOI:** 10.1186/s40164-025-00698-5

**Published:** 2025-09-30

**Authors:** Ilham Youssry, Nardeen Ayad

**Affiliations:** 1 Hematology and BMT Unit, Faculty of Medicine, Cairo University, Cairo, Egypt; 2 Faculty of Medicine, Cairo University Kasr Al-Ainy, Kasr Al Ainy Street, Cairo, Egypt

**Keywords:** Venous thromboembolism, Inherited thrombophilia, Next-generation sequencing, Genetic testing, Precision medicine

## Abstract

Venous thromboembolism (VTE) remains a significant cause of morbidity and mortality, particularly among individuals with inherited thrombophilia. Despite the widespread use of thrombophilia testing, its clinical value is often questioned due to inconsistent guidelines and limited prospective evidence. Traditional testing panels target only a narrow set of common variants—such as Factor V Leiden and Prothrombin G20210A—and may miss rare, complex, or combined mutations, especially in high-risk patients, including pediatric populations and those with unprovoked events or atypical presentations. This correspondence aims to re-evaluate the clinical role of thrombophilia testing in light of next-generation sequencing (NGS), a technology that offers a broader, more precise assessment of heritable thrombotic risk. We discuss how NGS improves variant detection, enhances risk stratification, and supports a precision medicine framework—particularly in clinical scenarios where standard algorithms fail. By integrating emerging evidence and real-world applications, we advocate for an updated, individualized approach to genetic testing in VTE care.

To the Editor,

Venous thromboembolism (VTE) continues to pose complex diagnostic and management challenges, particularly in cases of early-onset thrombosis, unusual anatomic locations, or strong familial clustering. While current clinical guidelines often discourage routine thrombophilia testing due to its perceived limited utility, this approach may overlook patients whose clinical presentation warrants a more nuanced, personalized evaluation. Inherited thrombophilia contributes significantly to recurrence risk, and next-generation sequencing (NGS) is reshaping the landscape of how we define, detect, and act upon that risk [1–3].

Traditional thrombophilia panels are typically limited to a handful of well-known variants, such as Factor V Leiden, Prothrombin G20210A, and deficiencies in protein C, protein S, or antithrombin. These tests are useful in straightforward scenarios but fall short when confronted with complex or atypical thrombophilic phenotypes [4]. In many patients—especially those with pediatric onset or a strong family history—conventional testing yields normal results despite suggestive clinical patterns.

Next-generation sequencing (NGS) expands the diagnostic lens beyond these constraints. It allows for the detection of rare single-nucleotide variants, structural rearrangements, copy number variants, and mutations in regulatory or noncoding regions [5, 6]. This technology is especially useful in revealing oligogenic or polygenic contributions to thrombosis, which often go undetected in standard workups. In pediatric settings, early identification of hereditary risk can significantly impact lifelong treatment decisions, reproductive counseling, and long-term surveillance strategies [7].

To rationalize testing decisions, we propose a zone-based decision framework (Fig. 1). This model stratifies patients based on the degree to which thrombophilia testing is likely to inform treatment decisions.

**Fig. 1:**
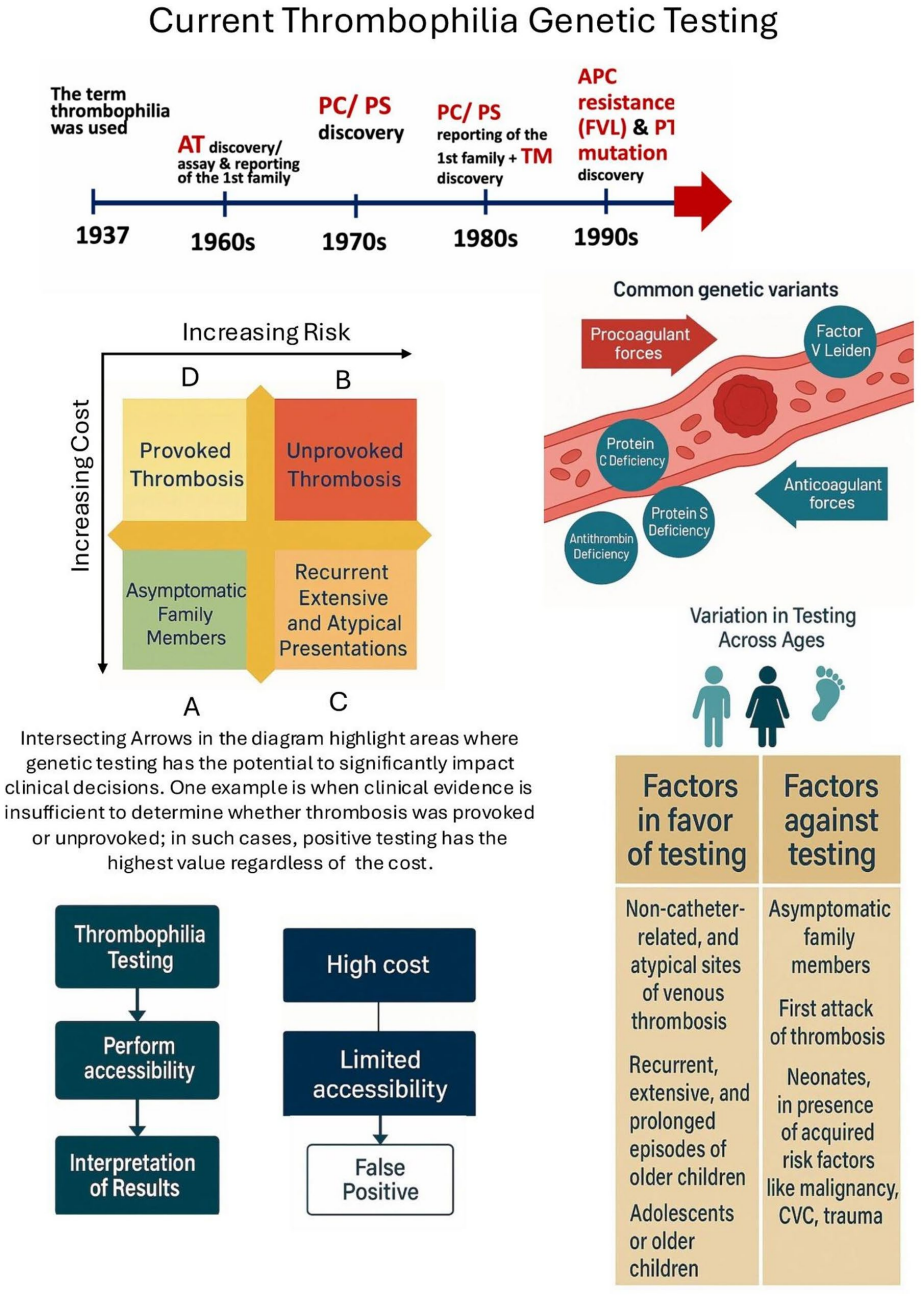
Decision Zones for Thrombophilia Testing. **Zone A**: Asymptomatic family members. Routine testing is generally not cost-effective, as results are unlikely to alter management. **Zone B**: Patients with unprovoked thrombosis. Testing may not affect the decision to treat but can guide the duration or intensity of anticoagulation. **Zones C and D**: Represent equivocal or intermediate-risk scenarios. Here, testing may sway decisions toward initiating or withholding treatment and may support more tailored prophylactic or surveillance strategies

There are several considerations for and against thrombophilia testing. When used selectively, testing can inform decisions on anticoagulation duration, support reproductive counseling, guide family screening, and provide psychological reassurance when a heritable predisposition is confirmed. However, its routine use remains debated, particularly when results do not impact acute management. Additional concerns include misinterpretation of variants, downstream anxiety, cost inefficiency in low-risk populations, and the potential for insurance or employment discrimination [8, 9].

With the increasing availability of genomic tools, next-generation sequencing (NGS) has gained traction as a strategy to expand and refine thrombophilia evaluation. Most commonly, NGS refers to targeted multigene panels designed to sequence a curated set of thrombosis-associated genes. Compared to traditional testing, which typically focuses on a handful of common variants, these panels offer a broader view, enabling detection of rare and novel mutations, including co-inherited variants under oligogenic models. In some cases, they may also detect splice-site and deep intronic variants, depending on panel design, and allow variant classification through integrated annotation pipelines.

To illustrate these evolving dimensions of thrombophilia testing and clarify the role of NGS, Table 1 provides a two-part overview. **Part A** highlights recent influential studies that have evaluated multigene sequencing strategies in thrombophilia, offering insight into how these approaches enhance variant detection while exposing interpretive gaps. **Part B** directly compares traditional thrombophilia testing with modern targeted NGS panels, focusing on capabilities most relevant to clinical decision-making. This combined framework emphasizes both the expanded diagnostic potential of NGS, and the caution required when translating complex genetic data into clinical care.

**Table 1:** Recent advances and diagnostic advantages of NGS for thrombophilia

A. Key Studies Using NGS in Thrombophilia Evaluation			
Study	Methodology	Key Findings	Implications
Verstraete et al. (2025) [10]	31-gene NGS panel in VTE patients	63% had genetic variants; 41% were undetected by conventional testing; 19% had variants in multiple genes	NGS expands variant detection, reveals potential oligogenic contributions
Seyerle et al. (2023) [11]	Whole genome sequencing in a large, multi-ethnic cohort	Confirmed rare variants in PROC and PROS1; filtering strategy affects gene discovery	Highlights complexity and limitations of rare variant interpretation
Ramanan et al. (2025) [12]	Review of multigene NGS panels	Emphasized improved diagnostic yield and interpretation challenges, particularly for VUS	NGS has value but requires expert interpretation and clinical context
**B. Comparison of Traditional Thrombophilia Testing vs. NGS-Based Targeted Gene Panels**			
**Traditional Thrombophilia Testing**		**NGS-Based Targeted Gene Panels**	
Targets 3–5 common variants (e.g., Factor V Leiden, Prothrombin G20210A)		Covers dozens of thrombosis-related genes, including rare and novel variants	
Misses noncoding, regulatory, or deep intronic mutations		Captures selected noncoding and splice-site variants within targeted regions	
Cannot detect structural or copy number variations		Can detect select structural variants and CNVs depending on panel design	
Limited to single-gene analysis		Supports oligogenic interpretation when multiple variants are present	
Binary classification (mutation present/absent)		Allows classification of variants of uncertain significance (VUS) using integrated annotation tools	

Beyond targeted panels, broader sequencing approaches such as whole-exome sequencing (WES) and whole-genome sequencing (WGS) offer more comprehensive genomic interrogation. WES captures all coding regions across the genome, while WGS extends coverage to noncoding sequences, structural variants, and other regulatory elements. These methods are particularly valuable in patients with atypical clinical presentations, negative panel testing despite strong family histories, or suspected complex inheritance. However, they come with trade-offs—higher cost, increased analytic complexity, and a greater chance of uncovering incidental or ambiguous findings. As such, they remain primarily reserved for research or difficult diagnostic scenarios rather than routine testing.

Further technical developments continue to expand the potential of genomic diagnostics. Long-read sequencing platforms, capable of analyzing DNA fragments tens of thousands of base pairs in length, enable continuous reads through regions that were previously difficult to sequence—such as repetitive or GC-rich areas. This enhances the detection of structural variants, repeat expansions, and complex rearrangements that may be missed by short-read methods [10]. In parallel, advances in bioinformatics—particularly those incorporating splicing prediction tools, variant annotation frameworks, and clinical-genomic databases—are improving our ability to interpret variants of uncertain significance, especially in regulatory or poorly characterized regions [13, 14].

As genomic testing becomes more accessible, its selective use in well-defined clinical decision zones—such as early-onset or unprovoked VTE, strong family history, or thrombosis in unusual locations—may help refine risk assessment, guide long-term care, and improve diagnostic confidence. In these contexts, targeted NGS panels can offer high diagnostic yield when supported by appropriate pre- and post-test counseling, expert review, and careful attention to the clinical context.

Ultimately, the incorporation of NGS into thrombophilia evaluation reflects a broader shift toward precision hematology. Moving beyond reflexive or binary testing strategies, clinicians are encouraged to adopt nuanced, individualized approaches that consider patient phenotype, likelihood of actionable results, and broader ethical implications. As evidence grows and technologies evolve, future guidelines will need to embrace a stratified, genomics-informed framework—one that aligns testing practices with patient-centered care and advances in molecular medicine.

## Data Availability

No datasets were generated or analysed during the current study.

## References

[1] Baglin T, Gray E, Greaves M, Hunt BJ, Keeling D, Machin S, et al. Clinical guidelines for testing for heritable thrombophilia. Br J Haematol. 2010;149(2):209–20. 20128794 10.1111/j.1365-2141.2009.08022.x

[2] Connors JM. Thrombophilia testing and venous thrombosis. N Engl J Med. 2017;377(12):1177–87. 28930509 10.1056/NEJMra1700365

[3] De Stefano V, Rossi E, Paciaroni K, Leone G. Screening for inherited thrombophilia: indications and therapeutic implications. Haematologica. 2002;87(10):1095–108. 12368166

[4] Middeldorp S. Inherited thrombophilia: a double-edged sword. Hematology. 2016;2016(1):1–9. 27913455 10.1182/asheducation-2016.1.1 PMC6142488

[5] Trégouët DA, Morange PE. Next-generation sequencing strategies in venous thromboembolism: in whom and for what purpose? J Thromb Haemost. 2024;22(7):1826–34. 38641321 10.1016/j.jtha.2024.04.004

[6] Zöller B, Svensson PJ, Dahlbäck B, Lind-Hallden C, Hallden C, Elf J. Genetic risk factors for venous thromboembolism. Expert Rev Hematol. 2020;13(9):971–81. 32731838 10.1080/17474086.2020.1804354

[7] Goldenberg NA, Bernard TJ. Venous thromboembolism in children. Hematol Oncol Clin North Am. 2010;24(1):151–66. 20113900 10.1016/j.hoc.2009.11.005

[8] Kujovich JL. Thrombophilia and pregnancy complications. Am J Obstet Gynecol. 2004;191(2):412–24. 15343215 10.1016/j.ajog.2004.03.001

[9] Middeldorp S, Nieuwlaat R, Baumann Kreuziger L, Coppens M, Houghton D, James AH, et al. American society of hematology 2023 guidelines for management of venous thromboembolism: thrombophilia testing. Blood Adv. 2023;7(22):7101–38. 37195076 10.1182/bloodadvances.2023010177 PMC10709681

[10] Verstraete A, De Vera MJ, Van Laer C, Van Thillo Q, Baert S, Kint C, et al. Multigene panel for thrombophilia testing in venous thromboembolism. J Thromb Haemost. 2025;23(6):1838–49. 39800257 10.1016/j.jtha.2024.12.041

[11] Seyerle AA, Laurie CA, Coombes BJ, Jain D, Conomos MP, Brody J et al. Whole Genome Analysis of Venous Thromboembolism: the Trans-Omics for Precision Medicine Program. Circulation: Genomic and Precision Medicine. 2023;16(2):e003532. 10.1161/CIRCGEN.121.003532 PMC10151032 36960714

[12] Ramanan R, Verstraete A, Van Laer C, Freson K. Implementation and clinical utility of multigene panels for bleeding, platelet, and thrombotic disorders. Journal of Thrombosis and Haemostasis [Internet]. 2025 May 8 [cited 2025 Jul 26]; Available from: https://www.sciencedirect.com/science/article/pii/S1538783625002776 10.1016/j.jtha.2025.04.026 PMC12212629 40345666

[13] Richards S, Aziz N, Bale S, Bick D, Das S, Gastier-Foster J, et al. Standards and guidelines for the interpretation of sequence variants: a joint consensus recommendation of the American college of medical genetics and genomics and the association for molecular pathology. Genet Med. 2015;17(5):405–24. 25741868 10.1038/gim.2015.30 PMC4544753

[14] Xue Y, Ankala A, Wilcox WR, Hegde MR. Solving the molecular diagnostic testing conundrum for Mendelian disorders in the era of next-generation sequencing: single-gene, gene panel, or exome/genome sequencing. Genet Med. 2015;17(6):444–51. 25232854 10.1038/gim.2014.122

